# Peculiar Properties of the La_0.25_Ba_0.25_Sr_0.5_Co_0.8_Fe_0.2_O_3−δ_ Perovskite as Oxygen Reduction Electrocatalyst

**DOI:** 10.3390/molecules28041621

**Published:** 2023-02-08

**Authors:** Chiara Aliotta, Maria Costa, Leonarda Francesca Liotta, Valeria La Parola, Giuliana Magnacca, Francesca Deganello

**Affiliations:** 1 Istituto per lo Studio dei Materiali Nanostrutturati, Consiglio Nazionale delle Ricerche, 90146 Palermo, Italy; 2 Dipartimento di Chimica, Università degli Studi di Torino, 10125 Torino, Italy

**Keywords:** heterostructure, oxygen vacancies, Ba-doped-LSCF, ORR, co-doping

## Abstract

The electrochemical reduction of molecular oxygen is a fundamental process in Solid Oxide Fuel Cells and requires high efficiency cathode materials. Two La_0.25_Ba_0.25_Sr_0.5_Co_0.8_Fe_0.2_O_3−δ_-based perovskite compounds were prepared by solution combustion synthesis, and characterized for their structural, microstructural, surface, redox and electrochemical properties as potential cathodes in comparison with Ba_0.5_Sr_0.5_Co_0.8_Fe_0.2_O_3−δ_ and La_0.5_Sr_0.5_Co_0.8_Fe_0.2_O_3−δ_ perovskites. Results highlighted that calcination at 900 °C led to a “bi-perovskite heterostructure”, where two different perovskite structures coexist, whereas at higher calcination temperatures a single-phase perovskite was formed. The results showed the effectiveness of the preparation procedures in co-doping the A-site of perovskites with barium and lanthanum as a strategy to optimize the cathode’s properties. The formation of nanometric heterostructure co-doped in the A-site evidenced an improvement in oxygen vacancies’ availability and in the redox properties, which promoted both processes: oxygen adsorption and oxygen ions drift, through the cathode material, to the electrolyte. A reduction in the total resistance was observed in the case of heterostructured material.

## 1. Introduction

In the present energy transition, alternative efficient energy generation is in demand, considering the urgent necessity of lowering carbon dioxide emission. It has been acknowledged that solid oxide fuel cells (SOFCs) provide clean energy with higher efficiency and a lower environmental impact than traditional combustion engines through the exploitation of renewable sources [1]. Today, the crucial issue for a reliable commercial diffusion of SOFC devices is represented by the capability of lowering the operation temperatures into the so-called intermediate temperature (IT) range (~500–750 °C) [2]. Unavoidably, lowering working temperatures entails a decrease in kinetic processes involving electrodes as electrochemical reactions and charge transport [3]. In the perspective of rendering IT-SOFCs competitive on the market, it is mandatory to design electrode materials with a suitable electrocatalytic activity and a mixed ionic-electronic conductivity. As far as cathode materials are concerned, today, one of the main challenges is represented by the concrete possibility of promoting the oxygen reduction reaction (ORR) through the specific and selective absorption of oxygen from the air [4]. Cathode materials promote catalytically and electrocatalytically the inclusion of adsorbed oxygen molecules in the lattice as oxygen ions (O^2−^), which are drifted along a cathode towards an electrolyte. For ensuring satisfying overall resistance at the cathode/electrolyte interface, oxygen anions should be easily incorporated into electrolyte lattices. In fact, oxygen anion formation and mobility require the presence of adequate oxygen lattice vacancy numbers and distribution [3]. Therefore, at an intermediate temperature an eligible electrocatalyst for oxygen reduction reaction has to be characterized by a fine tuning of the oxygen vacancies distribution on the first layers of the material’s surface, for an optimal oxygen exchange at the cathode/air interface, and in the bulk for ensuring oxygen anions’ transfer [3,5].

Several strategies have been adopted over the years to improve the cathode material performance and, from a green point of view, low cost and efficient cathode materials are advisable. Among them, perovskite-type oxides such as doped-lanthanum strontium ferro-cobaltite (La_1−x_Sr_x_Co_1−y_Fe_y_O_3−δ_, LSCF or La_1−x_Sr_x_Fe_1−y_Co_y_O_3−δ_, LSFC) and barium strontium cobaltite (Ba_0.5_Sr_0.5_Co_0.8_Fe_0.2_O_3−δ_, BSCF) have been widely investigated [5,6,7,8]. Lanthanum strontium ferro-cobaltite perovskites represent a good compromise between suitable electrocatalytic activity and mixed ionic-electronic conductivity. These cobalt-based materials are characterized by a rhombohedral perovskite type structure, and the co-presence of iron and cobalt at the B-site ensures a reasonable oxygen deficiency and good chemical compatibility with ceria-based electrolytes. In general, doping the A site with aliovalent ions positively affects electronic conductivity, decreasing electrode polarization making these materials adequate mixed ionic-electronic conductors (MIECs). In fact, a modulation of nature and a concentration of dopants increases the oxygen vacancies content in the material and improves the overall electrochemical performance. For example, recently Xie et al. [9] verified that doping La_0.6_Sr_0.4_Fe_0.8_Co_0.2_O_3−δ_ with barium gave rise to a reduction of polarization resistance with respect to samples doped with strontium only.

Shao and Haile [10], in 2004, proposed Ba_0.5_Sr_0.5_Co_0.8_Fe_0.2_O_3−δ_ as a new and compelling cathode material for solid oxide fuel cells properly in view of its high oxygen permeation generated by a considerable oxygen vacancy concentration. Upon 900 °C, BSCF has a cubic perovskite-type structure, rich in oxygen vacancies, whereas at about 800 °C, it undergoes a partial reversible transition to a hexagonal perovskite phase at the grain boundaries of the cubic phase, and this affects the overall cathode performance [11]. Several attempts have been made in the literature to stabilize the cubic perovskite-type structure and improve the mixed ionic–electronic conductivity by doping BSCF with different cations [12,13,14,15]. For example, Yang et al. [14] tested BSCF doped with 10 wt.% of cerium as a cathode material and observed the formation of a single cubic phase and a reduction of polarization resistance with respect to BSCF. Several years before, J. Kim et al. [15] proposed a La-doped BSCF, in detail La_0.5_Ba_0.25_Sr_0.25_Co_0.8_Fe_0.2_O_3−δ_, to stabilize the cubic structure after up to 300 h of air annealing at 700 °C. The authors concluded that La_0.5_Ba_0.25_Sr_0.25_Co_0.8_Fe_0.2_O_3−δ_ showed a superior electrical conductivity compared with BSCF as a function of both temperature and p(O_2_).

Another strategy adopted in the literature was mixing LSCF and BSCF materials to obtain a superior cathode able to bring peculiar features of each single structure [16]. Clematis et al. [17] showed that mixing commercial Ba_0.5_Sr_0.5_Co_0.8_Fe_0.2_O_3−δ_ and La_0.6_Sr_0.4_Fe_0.8_Co_0.2_O_3−δ_ by ball milling with different volume ratios, it is possible to tune material performance between 500–650 °C. The polarization resistance decreases by increasing the BSCF/LSCF volume ratio and, in general, is always larger than LSCF and BSCF as single phases. Recently, Dey et al. [18] synthesized heterostructured material starting from La_0.6_Sr_0.4_Co_0.8_Fe_0.2_O_3−δ_ and Ba_0.6_Sr_0.4_Co_0.8_Fe_0.2_O_3−δ_ powders. The authors referred to a decrease in resistance due to the optimization of oxygen vacancies in the heterostructure with respect to BSCF’s and LSCF’s single phases. The affirmation of the importance revested by heterostructure as way for improving the performance of oxide materials for energy devices is relatively recent. Like composite materials, heterostructures differ in the positive synergic effects, for example on catalytic or electrochemical features, due to the intimate connection between the different phases [19,20,21]. The aim of this work moves from the idea that a new promising electrocatalyst should exhibit the main properties of both LSCF- and BSCF-based electrocatalysts; namely, as referred above, appropriate mixed-ionic conductivity for IT-SOFCs working conditions and suitable oxygen permeation for ensuring a good oxygen exchange at intermediate temperature, respectively. To reach the scope, the possibility to improve the electrocatalytic oxygen reduction properties of an LSCF perovskite has been explored through the introduction of barium as a dopant. To the best of our knowledge, a perovskite oxide with the nominal composition La_0.25_Ba_0.25_Sr_0.5_Co_0.8_Fe_0.2_O_3−δ_ has not yet been investigated as evidenced by Table 1, where similar compositions containing barium are listed. According to the literature [19,20], it is possible to prepare metal oxide-based heterostructure with non-hierarchical arrangements as finely mixed compounds, at the nanometric level, through solution-based syntheses. Therefore, La_0.25_Ba_0.25_Sr_0.5_Co_0.8_Fe_0.2_O_3−δ_-based materials were prepared by solution combustion synthesis, which is a fast, reproducible, versatile and efficient methodology for the preparation of multicomponent perovskite-type oxides [22]. The use of SCS as way for preparing both single phase and heterostructure materials was accompanied with two different thermal treatments. For the sake of comparison, an LSCF (La_0.5_Sr_0.5_Co_0.8_Fe_0.2_O_3−δ_), and a BSCF (Ba_0.5_Sr_0.5_Co_0.8_Fe_0.2_O_3−δ_), were also prepared using the same experimental conditions. To investigate the effect of Ba introduction in both La_0.25_Ba_0.25_Sr_0.5_Co_0.8_Fe_0.2_O_3−δ_-based systems on the chemical physical properties and to evaluate the oxygen vacancies content and the capability of release oxygen, all the powders were characterized by X-ray Diffraction, Transmission Electron Spectroscopy, X-ray Photoelectron Spectroscopy, Thermo-Gravimetric Analysis and H_2_-Temperature Programmed Reduction techniques. The powders were deposited onto a Ce_0.8_Sm_0.2_O_2_ (SDC) electrolyte and half-cells were examined by Electrochemical Impedance Spectroscopy at various temperatures.

## 2. Results and Discussion

**Figure 1:**
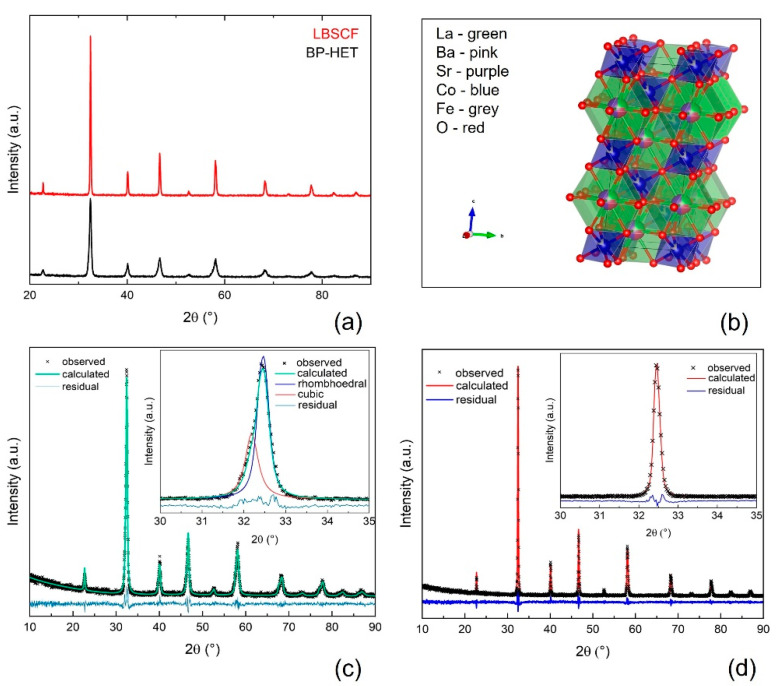
Panel (**a**): XRD patterns of BP-HET and LBSCF; Panel (**b**): Graphical representation of LBSCF phase extracted by Rietveld refinement; Panel (**c**): Graphical result of Rietveld refinement of BP-HET; black crosses: observed data; red line: calculated data; blue line: residual; Panel (**d**): Graphical result of Rietveld refinement of LBSCF; black crosses: observed data; red line: calculated data; blue line: residual.

The XRD patterns of the powders with the nominal composition La_0.25_Ba_0.25_Sr_0.5_Co_0.8_Fe_0.2_O_3−δ_ after thermal treatment at 900 °C (BP-HET) and at 1000 °C (LBSCF) for 5h are shown in Figure 1a. Both patterns were analyzed by the Rietveld method and the results of the refinements are listed in Table S1, whereas Figure 1c,d displays the relative graphical Rietveld refinements. The thermal treatment at 900 °C favors the formation of an in situ grown heterostructure made up of a main phase with a rhombohedral lattice similar to an LSCF (R-3c, PDF-00-049-0283, 77 wt.%), and a second phase with a cubic lattice similar to a BSCF (Pm-3m, PDF-00-055-0563, 23 wt.%). It is worth pointing out that any attempt made to fit the BP-HET pattern with a single cubic phase, or a single rhombohedral phase was totally unsuccessful. By comparing BP-HET cell lengths and cell volume with those of BSCF900 and of LSCF900 listed in Table S1 (see Supplementary Materials), it is evident that part of the Ba^2+^ (1.61 Å) ions entered the rhombohedral phase, causing an expansion of the rhombohedral unit cell, while it is likely that La^3+^ (1.36 Å) ions occupy the A-sites of the cubic phase, leading to a unit cell contraction. Interestingly, it seems that La-doping in the cubic BSCF-type phase of the heterostructure avoids the formation of the typical hexagonal impurity phase that was detected in BSCF900 (Table S1). Such results are not trivial and are intimately correlated to the synthesis process. A fundamental step of SCS is the formation of a gel where all the metal cations, the fuel and the additional oxidant are linked together in a cage network formed in situ as a result of the gradual water evaporation from the combustion mixture [22,24]. In a gel network, all the components are kept in specific positions, depending on the bonds formed between the fuel-complexant and the metal cations precursors, which have very low freedom to change their positions. During the fast increase in temperature, up to ca. 790 °C for few seconds, caused by the combustion of the gel, the organic part of the gel is burned out, the metal cations are forced to get closer and the germination of kinetically favored phases is promoted. Therefore, the gel acts as a footprint for the phase/s to be formed after combustion. A further heat treatment promotes the thermodynamic stabilization of the favorite phase. In BP-HET case, the presence of two perovskite oxides with a different lattice arrangement induces us to suppose that both phases are preferred until 900 °C, but the 77 wt.% of the rhombohedral phase hints at a major thermodynamic stability of this phase with respect to the cubic one. The analysis of LBSCF reveals that the specimen exhibits a single rhombohedral phase with larger lattice parameters than for LSCF1000 (Table S1). Thus, by introducing 25 wt.% of barium in the A-site of LSCF, the rhombohedral lattice distortion is retained. This confirms the previous observation regarding the major thermodynamic stability of the rhombohedral phase. Actually, after treatments at 1000 °C the cubic phase apparently disappears. However, the presence of Ba^2+^ ions, having higher ionic radius with respect to Sr^2+^ (1.44 Å), and La^+3^ (1.36 Å) causes an increase in rhombohedral unit cell volume. On the other hand, Szpunar et al. [25] attributed a cubic perovskite structure to Ba-doped lanthanum cobaltite with 25 wt.% of barium, highlighting the tendency, for pure cobaltite, to arrange in a cubic structure. Before, Kim et al. [15] observed a similar result for La_0.5_Ba_0.25_Sr_0.5_Co_0.8_Fe_0.2_O_3−δ_, while recently Xie et al. [9], studying La_0.4_Ba_0.2_Sr_0.4_Co_0.2_Fe_0.8_O_3−δ_ by coupled experimental and computational studies, noticed the formation of the rhombohedral lattice structure. In order to reconcile this evidence with our data, it is possible to hypothesize that, beside differences in chemical compositions, different oxidation state of B-site ions in LBSCF contribute to a distortion of the lattice from cubic to rhombohedral. In addition, looking at the unit cell volumes of LBSCF and of BP-HET, it is possible to argue that the rhombohedral phase present in the heterostructure is rich in Ba^2+^, likely rather close to the nominal composition of 25 wt.%. This result is confirmed by the LSCF1000 volume that is much lower than that of the LSCF-type phase in the heterostructure.

**Figure 2:**
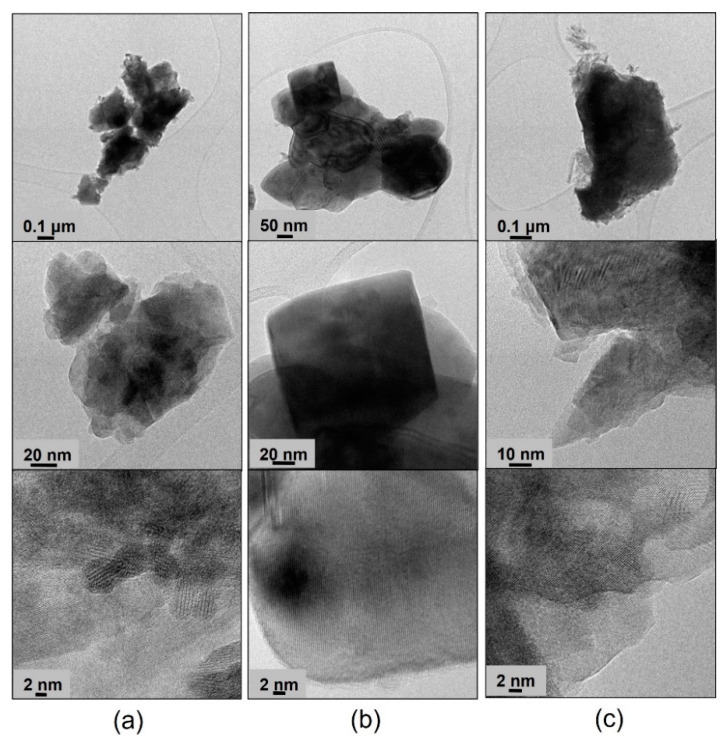
Low-(up), medium-(middle) and high-(down) resolution TEM images of BP-HET (**a**), LSCF900 (**b**) and BSCF900 (**c**).

**Figure 3:**
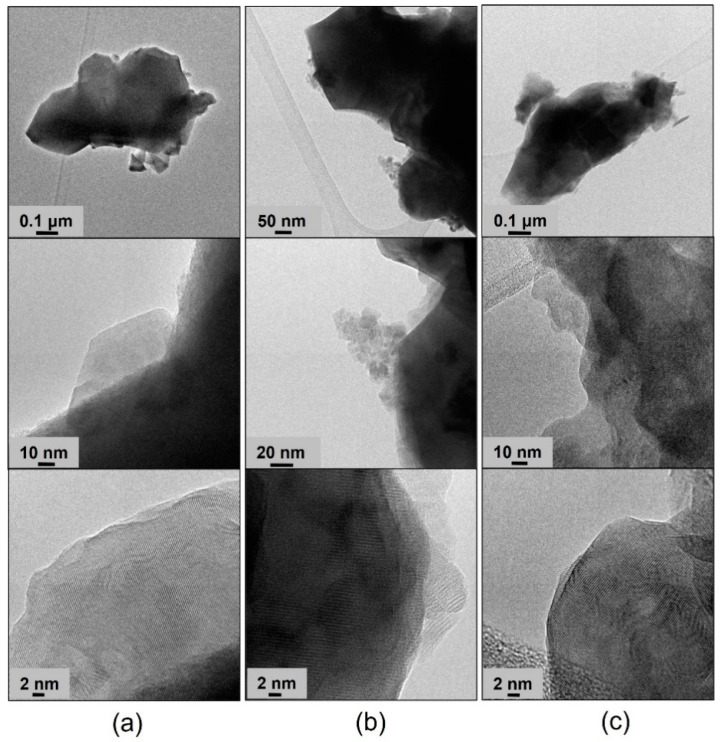
Low-(up), medium-(middle) and high-(down) resolution TEM images of LBSCF (**a**), LSCF1000 (**b**) and BSCF1000 (**c**).

The TEM images of BP-HET, LBSCF, LSCF and BSCF treated at 900 °C and 1000 °C at low, medium and high resolutions are reported in Figure 2 and Figure 3, respectively. Looking at the BP-HET micrographs (Figure 2a), the presence of aggregates of nanometric particles of about 20 nm with a rather rounded shape is evident. Extended and thick particles are also visible, but their scarce transparency made it impossible to recognize their crystalline arrangement. However, in the regions where the thickness of the particles made possible the observation, the most frequently measured d_hkl_ value corresponded to about 2.22 Å (see Supplementary Materials for detailed procedure). The same d_hkl_ value was observed for the LSCF900 sample, which was composed by extended, almost cubic, crystalline particles of about 100 nm of size. Such finding is in agreement with the fact that BP-HET contains 77 wt.% of rhombohedral phase. Apart from the 2.22 distance, other larger d_hkl_ values can be recognized in the BP-HET micrographs, and even if they are not directly relatable to XRD reflections, the observed enlargement of the value could be due to the insertion of some large barium ions in the rhombohedral lattice of LSCF. The insertion of Ba^2+^ seems to be gradual and variable, as a very wide series of d_hkl_ values can be observed in the BP-HET sample. On the other hand, the BSCF900 sample shows small dispersed acicular particle aggregates at the surface of extended ordered bigger crystals. In this case, a preferential disposition of the crystals allowing the recognition of one specific family of crystalline planes is not observable, as several d_hkl_ values from 1.70 to 3.10 Å can be measured in the micrographs. All the samples treated at 1000 °C (Figure 3) are made of more extended crystalline particles. LBSCF appears morphologically different compared with BP-HET and shows the d_hkl_ value of 2.22 Å, together with several other distances in the range of 2.2–3.1 Å, compatible with the gradual and variable inclusion of barium ions in the LSCF lattice, as described above. No significant changes related to the observed particle size and d_hkl_ of LSCF1000 with respect to LSCF900 were observed. In the case of BSCF1000, a specific family of crystalline planes characterized by a d_hkl_ value of 3.10 Å was often measured and no acicular particles were present, probably due to the disappearance of the hexagonal phase after the thermal treatment at 1000 °C. It is worth noting that BP-HET has a smaller particle size (20 nm) than its parent LSCF900 structure without Ba (100 nm). This result highlights that nucleation and the growing of the LSCF-type phase was affected by the contemporaneous nucleation and growing of the BSCF-type phase in the BP-HET, leading to highly nanostructured particles. In addition, although the extreme variability observed in d_hkl_ values, Figure 2 and Figure 3 allow for the direct observation of the crystalline features of BP-HET and LBSCF, as in the first case the material is constituted by aggregates of nanocrystallites among which the contact appears clearly, even if a heterojuction cannot be clearly evidenced, whereas in the second case a more extended crystalline structure, with a clear suppression of the possible interphases heterojunctions, can be observed.

**Figure 4:**
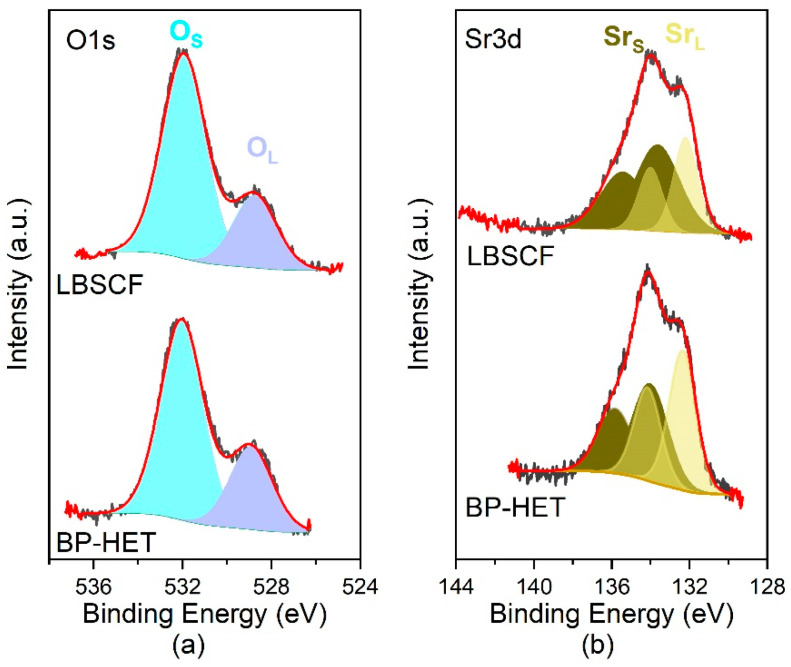
High resolution XPS O1s (**a**) and Sr 3d (**b**) region, respectively. Experimental signals (black lines), fitted model (red line), deconvolution O signal (light-blue and purple areas) and deconvolution Sr signal (green and yellow areas).

XPS analysis was performed with the intention of evaluating the surface state spread along the first layers of surface, which undeniably affect the oxygen adsorption process. The XPS spectra were recorded on the freshly annealed powders, to minimize the influence of physisorbed species. It is worth underlining that regions corresponding to Ba, Co, and Fe were not investigated for the following reasons: (i) Fe 2p region exhibited a low intensity signal due to low nominal 20 wt.% present in all samples; (ii) Co 2p and Ba 3d regions were overlapped leading to erroneous interpretations. Special attention was paid to O 1s and Sr 3d regions to evaluate differences on these surface states that are correlated with oxygen vacancy content and distribution [26]. As depicted in Figure 4, both regions show the presence of two components: a low energy component, O 1s = 528.5–529.0 eV and Sr 3d5/2 = 132.2 eV (Sr3d3/2 = 134 eV), attributed to the near-surface region of the perovskite lattice and which is indicated as a “lattice” component (O_L_-Sr_L_); a higher energy component, O 1s 532.2eV and Sr 3d5/2 = 133–134 eV (Sr3d3/2 = 135–135 eV), associated to perovskite surface termination and/or surface secondary phase, here indicated as “surface” component (O_S_-Sr_S_) [26,27,28]. The O 1s region is well fitted with two components at ca. 529 eV and ca. 532 eV, respectively (Figure 4a), whereas the O1s binding energies and relative percentages are listed in Table 2. The relative variations between the lattice and surface oxygen components may be indicative of different concentrations of oxygen vacancies and, in general, a decrease in the component at a low binding energy with respect to the component at a high binding energy is attributed to an increase in oxygen vacancies [29,30]. LBSCF exhibits a slightly higher surface to O 1’s contribution, and its first layers have a modestly higher amount of oxygen vacancies. On the other hand, the relative percentages of the “surface” and “lattice” components notably varied between the different compositions (Table S2). BP-HET and LBSCF show a relative percentage of the lattice components lower than LSCF samples and higher than BSCF ones. Therefore, barium doping causes an increase in surface oxygen vacancies with respect to LSCF, even though BSCF apparently remains characterized by a higher content of static oxygen surface vacancies as suggested by O 1’s relative percentage. An additional clue of the trend for oxygen vacancies is given by the analysis of the Sr 3d region. Looking at Table 2, it is evident that LBSCF is characterized by a higher relative percentage of surface Sr contribute with respect to BP-HET. According to Kuyyalil et al. [26], this behavior could be due to a surface enrichment in oxygen vacancies which acts as a driving force towards Sr ions which move to the surface. This also confirms the O 1’s results that outline that LBSCF is characterized by a higher amount of oxygen surface vacancies. However, a high level of Sr in an oxygen-rich atmosphere causes the formation of strontium oxide-based compounds that could affect negatively the electrocatalytic activity toward ORR [9,13,26]. Taking into account these considerations and comparing relative percentages also in the cases of BSCF and LSCF samples (see Table S2), it is possible to argue that BP-HET represents a good compromise with respect to other studied samples, since it shows a lower amount of Sr compared with LBSCF and other samples, but a higher amount of oxygen vacancies with respect to LSCF without Ba. In other words, co-doping with Ba and Sr positively affects the static oxygen vacancy amount in a LSCF perovskite.

**Figure 5:**
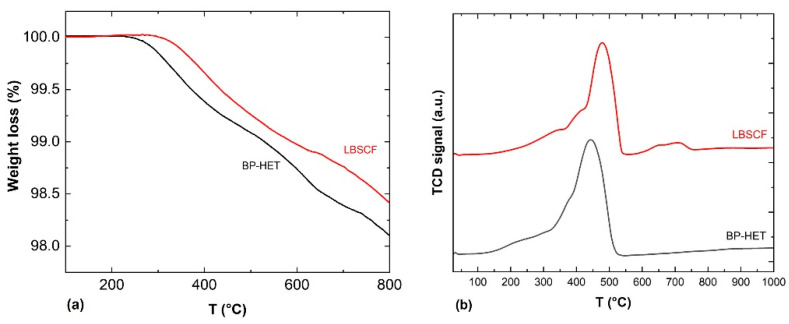
Panel (**a**): TGA curves calculated from Step 4 (see experimental part) for BP-HET (black line), and LBSCF (red line). Panel (**b**): H_2_-TPR profiles for BP-HET (black line), and LBSCF (red line).

Considering that the amount of vacancies affects both oxygen molecule adsorption and oxygen ion drift along the width of cathode [31], TGA experiments were performed to get further insights into the oxygen vacancies content of the examined samples. The weight loss percentages as a function of temperature calculated from Step 4 of the experiment are displayed in Figure 5a, while the weight loss values are listed in Table 3. In agreement with the existing literature, perovskite-type oxides present two type of chemisorbed oxygen species named as α and β. During the TPD-Step 4 experiment, the α-species are released between ~200–600 °C depending on sample composition and “suprafacial” oxygen vacancies content. The β-species, instead, desorb at a higher temperature, above 600 °C, because they are “interfacial or bulk” oxygen species associated with the reducible ions present in the perovskite-type oxide [24,32,33]. Looking at TGA curves, it appears clear that BP-HET is more prone to release oxygen than LBSCF, and this means that the heterostructure is characterized by a higher number of available oxygen species. In light of these findings and the XPS consideration about Sr surface enrichment, it is likely that LBSCF tends to form SrO in an oxygen-rich environment, blocking the available oxygen vacancies, even though it is characterized by a major amount of oxygen vacancies [26]. This translates into a lower ability to release oxygen with respect to BP-HET. To discriminate the role of Ba dopant, TGA measurements have been carried out also for BSCF and LSCF samples treated at 900 °C and 1000 °C (Figure S1). In the whole temperature range, all samples exhibit a different desorption behavior, hinting at a diversified presence of both α- and β-species. Below 600 °C BP-HET and LBSCF show the highest desorption and thus the highest defectivity. As summarized in Table 3 for each given compositions, samples treated at 1000 °C release less oxygen compared with 900 °C counterparts. This behavior is to be expected considering that a thermal treatment at higher temperatures promotes a greater coalescence of grains and therefore a reduction of oxygen vacancies. From these observations and by considering that all samples contain the same B-site composition, it is possible to argue that the amount of chemisorbed/desorbed oxygen is mainly determined by A-site doping. In other words, the different ratio between La-Ba/Sr inevitably influences the entire defectivity of the powder and, in this perspective, the co-doping of A site with Sr and Ba seems to create an optimum for perovskite lattice defects, especially in BP-HET that shows the highest vacancies content.

A further insight into oxygen ions’ mobility and the amount of oxygen released was carried out through H_2_-TPR measurements, performed from room temperature up to 1050 °C. Figure 5b shows TPR profiles for BP-HET and LBSCF. It is worth underlining that the only reducible species present in the studied samples are cobalt and iron. The BP-HET reduction profile shows a single enlarged peak that spans from ~100 °C to 500 °C centered at 440 °C with a shoulder below 300 °C, while for LBSCF it is possible to recognize two zones: the peak between ~200–550 °C, centered at 478 °C, and a small peak between 550–750 °C. The total hydrogen consumption, evaluated by applying a proper calibration curve to the TPR peaks, are c.a. 143 and 129 cm^3^/g, respectively, for the BP-HET and LBSCF specimens. These results are consistent with TGA data and confirm that BP-HET is richer in reactive oxygen species than LBSCF. However, TPR results evidenced that BP-HET also has a higher reducibility compared with the single-phase sample. As referred above, BP-HET is formed by two intimately mixed phases with rhombohedral and cubic structures, respectively, thus the presence of two different lattice arrangements positively influences the oxygen species’ reactivity.

**Figure 6:**
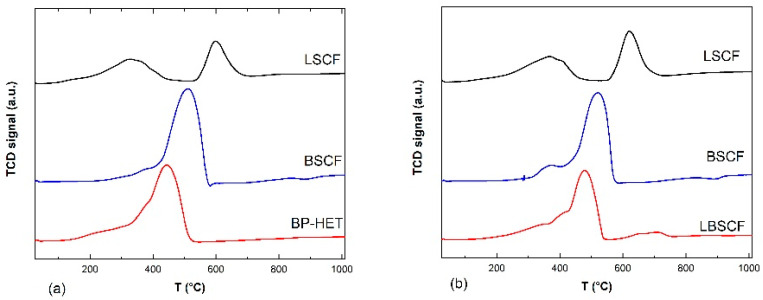
H_2_-TPR profiles of BP-HET, BSCF900 and LSCF900 (**a**) and LBSCF, BSCF1000 and LSCF1000 (**b**) between RT-1000 °C.

Therefore, to discern the correct binomial reduction processes-reduction peaks it is necessary to compare BSCF900 and LSCF900 (Figure 6a). Promptly, it can be easily noted that the BP-HET signal apparently resembles the BSCF900 profile characterized by a single asymmetric peak (~200–570 °C) centered at 510 °C with a shoulder peaked at 380 °C. According to the literature, the main reduction peak of BSCF900 is due to both Co^3+^ to Co^2+^ and Co^2+^ to Co^0^, whereas the shoulder at low temperature is ascribable to Co^4+^/Fe^4+^ to Co^3+^/Fe^3+^ [34]. In addition, following the considerations of Garcia-Lopez et al. [35] for La_1−x_Sr_x_Co_1−y_Fe_y_O_3−δ_ perovskite-type oxides, the enlarged lower peak, extended between 100–500 °C with a shoulder at about 160 °C, is due to the simultaneous reduction of Co^3+^ to Co^2+^ and Fe^3+^ to Fe^2+^, while the higher peak, laying between 550–700 °C with a maximum at 600 °C, is due to Co^2+^ to Co^0^. The reduction Fe^2+^ to Fe^0^ requires temperatures above 800 °C. In light of these attributions, it is reasonable to associate similar reduction processes for BP-HET, even though the whole composite profile is shifted towards lower temperatures hinting at the presence of more reducible cobalt and iron species and more mobile oxygen ions. It is likely that co-doping at A-site and the co-presence of two phases play a beneficial role in the mobility of the lattice oxygen ions. Considering the single-phase cases in Figure 6b, LBSCF seems to merge BSCF1000 and LSCF1000 profiles. In fact, it is possible to recognize two zones: the peak between ~200–550 °C resembles the BSCF1000 peak profile, while the small peak between 550–750 °C lies in the same zones of LSCF1000 second reduction peak. As evidenced by Figure 5b, LBSCF resembles BP-HET in the main peak, and, thus, the same assignation could be done. Furthermore, in this case the beneficial effect of co-doping at the A site is evident.

**Figure 7:**
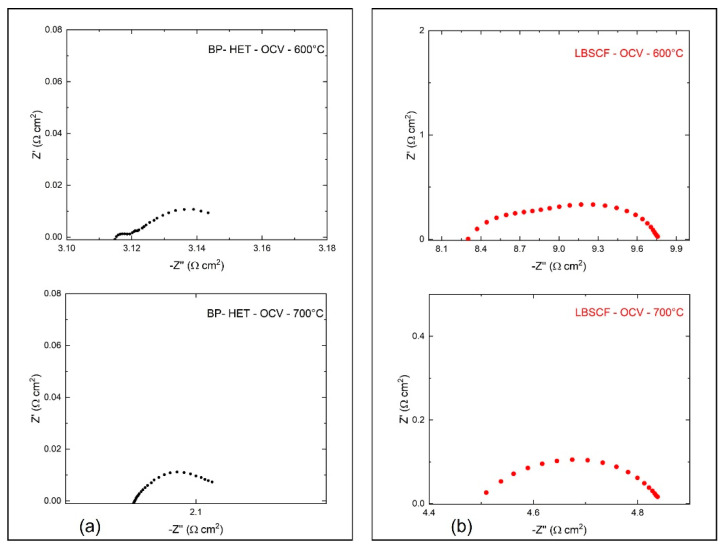
Impedance spectra acquired at 600 °C and 700 °C in air for BP-HET (black dots (**a**)) and LBSCF (red dots (**b**)).

**Figure 8:**
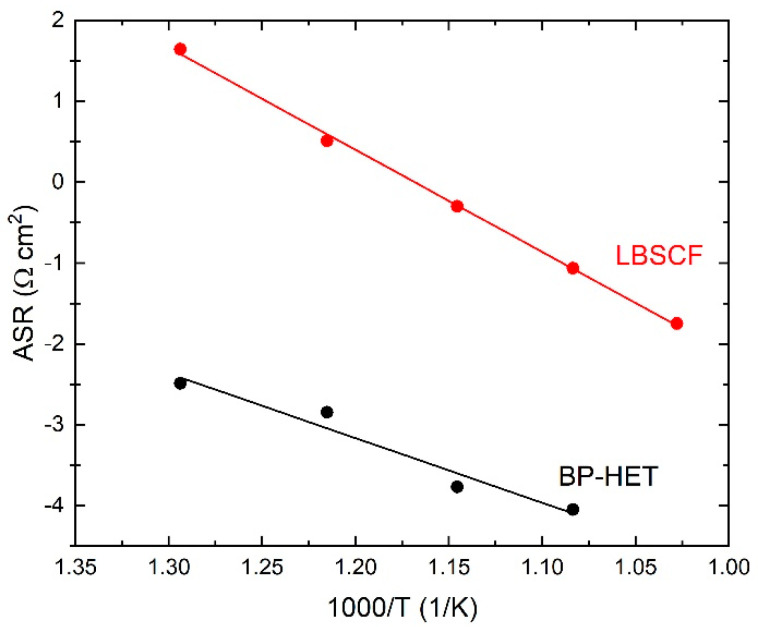
Area Specific resistance (ASR) for BP-HET (black line) and LBSCF (red line).

Electrochemical impedance spectroscopy was used to evaluate BP-HET and LBSCF assembled in half-cells over the 500–750 °C temperature range, under static air condition at Open Circuit Voltage (OCV). Figure 7 depicts the impedance spectra acquired for BP-HET and LBSCF at 600 and 700 °C. According to the literature, a complete equivalent circuit model for these systems, in the simple case, consists of three RQ circuits connected in series, where R is a resistance and Q is a constant phase element (CPE) defined as C = (R^1−nQ^)^1/n^ (C is the capacitance and n an additional fitting parameter) [15]. In details, the following contributions should be detected: (i) at high frequencies the contributions of electrolytes, (ii) at medium frequencies the electrode semicircle responsible of electrode/electrolyte interface, and (iii) at low frequencies the electrode transport that arises from surface oxygen exchange and charge bulk storage [16]. For the examined working temperatures, all these semicircles could be not explicitly evidenced in the impedance spectra. Therefore, only the total contribution at low and medium frequencies was evaluated. Total electrode resistance (R) is correlated to surface activity at interface air/electrode and to charge transfer along the cathode. Figure 7 indicates that the R value at 600 °C and at 700 °C is clearly lower for BP-HET than for LBSCF. Area Specific Resistance (ASR) results for both samples are reported in Figure 8, indicating that ASR was much lower for the BP-HET that for the LBSCF and confirming that the coexistence of two co-doped in A-site perovskite phases with different structures, as evidenced by XRD results, can promote oxygen adsorption and drift along cathode material under an electrochemical regime. These findings are in line with results evidenced by XPS and TGA tests, which indicate that an LBSCF sample is characterized by higher oxygen vacancies and strontium amounts on the surface, but in oxygen-rich environments these features translate into a reduction of oxygen vacancy availability with respect to BP-HET. As discussed previously, it is likely that, during EIS experiments in air, SrO-based compounds were formed on LBSCF, causing a detrimental effect on its conductivity. A decrease in the resistance was already reported by Clematis et al. [17] for an LSCF-BSCF micro composite, where the two phases were in contact only at micrometer level. In the work of Dey et al. [18], a special synthesis procedure was applied to obtain a LSCF-BSCF heterostructure with lower resistance than the parent LSCF and BSCF structures. In this work, the LSCF-type and BSCF-type phases were generated by a very simple in situ synthesis, the two phases are at nanometric contact, and this likely brings more synergy between the phases in the ORR process.

## 3. Materials and Methods

La_0.25_Ba_0.25_Sr_0.5_Co_0.8_Fe_0.2_O_3−δ_, Ba_0.5_Sr_0.5_Co_0.8_Fe_0.2_O_3−δ_ and La_0.5_Sr_0.5_Co_0.8_Fe_0.2_O_3−δ_ powders were prepared by solution combustion synthesis using La(NO_3_)_3_·6H_2_O (99.99% Sigma-Aldrich, Mailand, Italy), Ba(NO_3_)_2_ (99.99% Sigma-Aldrich), Sr(NO_3_)_2_ (≥99.99% Sigma–Aldrich) Co(NO_3_)_2_·6H_2_O (99.99% Sigma-Aldrich and Fe(NO_3_)_3_·9H_2_O (99.99% Sigma–Aldrich) as metal precursors and polyethylene glycol (PEG, MW 20000, Fluka Chemika) mixed with sucrose (Eridania Italia S.p.A., Bologna, Italy) in 1:1 ratio as combustion fuel mixture. The combustion reactions were carried out under over stochiometric condition, a reducers-to-oxidizers ratio (Φ) = 2.2, with a sucrose-to-metal cations molar ratio equal to 2. To achieve this Φ value, ammonium nitrate (NH_4_NO_3_, ≥99.0% Sigma–Aldrich) was added to the reaction mixture as oxidant additive. Metal nitrates, fuel and additives were dissolved in aqueous solution in a stainless-steel beaker, and the solution was magnetically stirred at 80 °C to form the gel. By increasing the temperature of the hot plate to about 220 °C, a self-sustaining combustion process was initiated, leaving a grey–black powder. Powders were fired in static air at 900 °C and at 1000 °C for 5 h. La_0.25_Ba_0.25_Sr_0.5_Co_0.8_Fe_0.2_O_3−δ_ obtained after firing at 1000 °C/5 h was named after LBSCF, the bi-perovskite heterostructure with nominal composition. La_0.25_Ba_0.25_Sr_0.5_Co_0.8_Fe_0.2_O_3−δ_, obtained after firing at 900 °C/5 h, was named after BP-HET, whereas Ba_0.5_Sr_0.5_Co_0.8_Fe_0.2_O_3−δ_ and La_0.5_Sr_0.5_Co_0.8_Fe_0.2_O_3−δ_ were named after BSCF900 and BSCF1000 or LSCF900 and LSCF1000, respectively, depending on the firing temperature used.

Membrane electrode assemblies (MEA) with symmetric cell configuration were assembled to carry out electrochemical impedance spectroscopy measurements. The electrolyte Ce_0.8_Sm_0.2_O_2−x_ (SDC) powder was synthesized according to a solution combustion synthesis procedure reported elsewhere [24]. The SDC powder was first treated in static air at 500 °C for 5 h and then isostatically pressed at 35 KPa in the form of pellets and sintered in static air at 1250 °C for ten hours. The final sintered pellets had a diameter (d) of about 10.5 mm, a thickness (t) of about 0.95 mm. The cathode materials were grinded with ethanol (99.8% Sigma-Aldrich) and with Polyethylene-Polypropylene Glycol (Pluronic^®^ F-127 Sigma-Aldrich) and mixed for 30 min in an ultrasonic bath to obtain a well homogenized suspension. The slurries were deposited by screen printing over both faces of sintered pellets, dried at 160 °C for 1 h for each face, and finally treated at 900 °C for 2 h in static air.

X-ray diffraction (XRD) measurements were collected with a Siemens D5005 X-ray powder diffractometer equipped with a curved graphite monochromator on the diffracted beam. The observed range 10–90° 2θ was scanned with a step size of 0.03° 2θ and an integration time of 3 s per step. Rietveld refinement of the diffraction patterns was carried out by using the GSAS-II package version 5365 [36]. In the structure refinement, where Chebyschev polynomials were chosen for the background profile, background coefficients, lattice constants, atomic coordinates, scale factors and crystallite size parameters were considered as variables. Database PDF-4+ 2022 (database version 4.2201, software version 4.22.0.2) released by ICDD was used for qualitative structural analysis.

TEM investigation was carried out by means of JEOL JEM 3010-UHR, acceleration voltage 300 kV. The powdery samples were dry dispersed on holed carbon film deposited on Cu grid.

X-ray photoelectron spectroscopy analyses were performed with a VG-Microtech ESCA 3000Multilab, equipped with a dual Mg/Al anode. The spectra were excited by the unmonochromatized Al Kα source (1486.6 eV) run at 14 kV and 15 mA. The analyzer operated in the constant analyzer energy (CAE) mode. For the individual peak energy regions, a pass energy of 20 eV set across the hemispheres was used. Survey spectra were measured at 50 eV pass energy. The sample powders were analyzed as powder, mounted on a double-sided adhesive tape. The pressure in the analysis chamber was in the range of 10^−8^ mbar during data collection. The invariance of the peak shapes and widths at the beginning and at the end of the analyses ensured absence of differential charging. Analyses of the peaks were performed with CasaXPS software, based on non-linear least squares fitting program using a weighted sum of Lorentzian and Gaussian component curves after background subtraction according to Shirley and Sherwood [37,38]. Atomic concentrations were calculated from peak intensity using the sensitivity factors provided with the software. The binding energy values are quoted with a precision of ±0.15 eV and the atomic percentage with a precision of ±10%.

Thermogravimetric analyses (TGA) were performed with a TGA/DSC1 STAR system Mettler Toledo. The sample (10 mg) was pretreated in N_2_ (99.999% Rivoira, 30 mL min^−1^) at 750 °C for 1 h (step 1), then it was cooled down to room temperature in air (99.999% Rivoira, 30 mL min^−1^) purified from CO_2_ and H_2_O (step 2) and purged with N_2_ (99.999% Rivoira, 30 mL min^−1^) for 15 min (step 3). After that, the sample was again treated under N_2_ (99.999% Rivoira, 30 mL min^−1^) by heating (rate 5 °C/min) from room temperature up to 1000 °C (step 4). Step 1 was performed to remove any adsorbed water, oxygen or carbonate species; step 2 aimed to fill by oxygen all the vacancies; step 3 aimed to eliminate all the physisorbed oxygen species; step 4, during which the removal of chemisorbed oxygen species occurred, was taken into account in order to evaluate the oxygen vacancies content of the sample. The evolution of gaseous species occurring during the above four steps was monitored by on line mass quadrupole (ThermostarTM, Balzers).

Reduction properties of the oxides were studied by temperature programmed reduction (TPR) measurements in H_2_/Ar (5%, 30 mL/min) in the range between room temperature and 1050 °C (heating rate 10 °C/min). Experiments were carried out with a Micromeritics Autochem 2910 instrument equipped with a thermal conductivity detector (TCD) for the evaluation of hydrogen consumption through the use of proper calibration curve. All powders (~0.1 g) were pre-treated in O_2_/He (5%, 30 mL/min) at 400 °C for 1 h, in order to remove any impurity, and then cooled down under inert atmosphere (Ar flow).

Complex impedance spectroscopy from 10^7^ to 10^−1^ Hz was carried out between 500 and 750 °C employing Methrohm Autolab impedance analyzer (AC voltage of 0.3 V) equipped with a ProboStat from NorECs. After 15 h of equilibration at 750 °C in static air, the impedance spectra were collected from symmetrical cells between 750–500 °C (50 °C steps, with 1 h equilibration at each temperature), and analyzed with Nova software version 2.1.5 released by Methrohm Autolab.

## 4. Conclusions

A La_0.25_Ba_0.25_Sr_0.5_Co_0.8_Fe_0.2_O_3−δ_ perovskite has been obtained by solution combustion synthesis and investigated for its chemical–physical properties, with the scope of tuning oxygen vacancies content and availability for its application as an electrocatalyst for oxygen reduction reaction in the intermediate temperature range. The positive effect of Ba was assessed, and a peculiar behavior was evidenced upon thermal treatment. Treatment at 1000 °C in air leads to a complete incorporation of barium ions into the rhombohedral perovskite structure, with a positive effect on the oxygen vacancies distribution, if compared with the analogous perovskite without barium. Treatment at 900 °C in air favors the formation of a bi-perovskite heterostructure, where a lanthanum-enriched BSCF-type cubic phase and a barium-enriched LSCF-type rhombohedral phase coexist. Our results highlighted the potential of a one-pot solution combustion synthesis in producing nanostructured perovskite-type heterostructures with improved properties, as evidenced by temperature programmed reduction, thermal gravimetric analysis and electrochemical impedance spectroscopy. The bi-perovskite heterostructure exhibits available oxygen vacancies on the surface and in the bulk and a lower area specific resistance for the oxygen reduction reaction with respect to the single-phase perovskite with the same nominal composition. Other literature findings on perovskite-type compounds were confirmed and more information on the perovskite-type heterostructures for clean energy applications was added, discussing the effect of the bi-perovskite heterostructure on the structural properties, redox properties and oxygen vacancies. The proposed strategy appears to be an intriguing way for the fine-tuning of electrode materials for IT-SOFCs.

## Figures and Tables

**Table 1:** List of Ba-doped materials.

Doping A-B Sites	Compositions	Reference
Ba, Sr-Co	La_0.6−x_Ba_x_Sr_0.4_Co_0.2_Fe_0.8_O_3−δ_ (x = 0.1, 0.2)	[9]
Ba, Sr-Fe	La_0.5_Ba_0.25_Sr_0.25_Co_0.8_Fe_0.2_O_3−δ_	[15]
Ba, La-Co	(Ba/La)_0.6_Sr_0.4_Co_0.8_Fe_0.2_O_3−δ_	[18]
Ba, Sr-Co	La_0.6−x_Ba_x_Sr_0.4_Co_0.2_Fe_0.8_O_3−δ_ (x = 0.05, 0.10, 0.15, 0.20)	[23]

**Table 2:** XPS data of BP-HET and LBSCF in terms of O 1s and Sr 3d5/2 binding energies and the relative percentage of the different component are given.

Sample	O 1s BE (eV), Relative Percentage (%)	Sr 3d5/2 BE (eV), Relative Percentage (%)
BP-HET	528.9, 29 532.0, 71	132.3, 53 134.0, 47
LBSCF	528.7, 26 531.9, 74	132.2, 38 133.6, 62

**Table 3:** Summary of TGA experiments per gram of powder.

Sample	Weight Loss Percentage %
BP-HET	1.90
LBSCF	1.61
LSCF900	1.16
LSCF1000	1.09
BSCF900	0.81
BSCF1000	0.80

## Data Availability

All the data are included both in the article and in the supporting information.

## Supplementary Materials

The following supporting information can be downloaded at: https://www.mdpi.com/article/10.3390/molecules28041621/s1, Figure S1: TGA curves for all samples; Table S1 Results of Rietveld Refinement; Table S2: Results of XPS Analysis; Paragraph: Image analysis approach: HRTEM results.

## Sample Availability

Samples are available from the authors.
