# Supplementary material for: Peculiar Properties of the La0.25Ba0.25Sr0.5Co0.8Fe0.2O3−δ Perovskite as Oxygen Reduction Electrocatalyst
Source: Molecules. 2023 Feb 8;28(4):1621. doi: 10.3390/molecules28041621 (PMC9965584; doi:10.3390/molecules28041621)
Supplement: Supplementary file 1 [file molecules-28-01621-s001.zip › molecules-2149608-supplementary.pdf]

Supplementary Materials

Table S1: Results of Rietveld Refinement

Table S1: Label, space group, weight phase fraction (wt.%) with relative volume (V) and lattice parameters (a, b, c) of BP-HET, LBSCF, BSCF900, BSCF1000, LSCF900 and LSCF1000. Uncertainty is on the last digit for volumes and cell parameters; uncertainty for wt.% is ≤3%.

Samples	Space group	a (Å)	b (Å)	c (Å)	V ($Å^3$)	wt.%	χ^2	R^{F2}(%)
BP-HET	R-3c	5.48595	5.48595	13.36251	348.275	77	1.34	10.03
	Pm-3m	3.90357	3.90357	3.90357	59.482	23		
LBSCF	R-3c	5.48328	5.48328	13.39569	348.801q	100	1.28	10.18
BSCF900	Pm-3m	3.98612	3.98612	3.98612	63.336	96	1.20	16.87
	P63-mc	11.71647	11.71647	6.90845	821.311	4		
BSCF1000	Pm-3m	3.98435	3.98435	3.98435	63.252	100	1.23	21.02
LSCF900	R-3c	5.43196	5.43196	13.26246	338.897	100	1.42	7.93
LSCF1000	R-3c	5.43164	5.43164	13.25898	338.769	100	1.26	8.71

Table S2: Results of XPS Analysis

Table S2: XPS data of BSCF900, BSCF1000, LSCF900 and LSCF1000 in terms of O1s and Sr 3d5/2 binding energies and the relative percentage of the different component are given.

Samples	O1s BE (eV), relative percentage (%)	Sr 3d5/2 BE (eV), relative percentage (%)
BSCF900	529.4, 24	133.5, 100
	532.2, 76	
BSCF1000	529.2, 22	133.6, 100
	531.7, 78	
LSCF900	528.8, 32	132.3, 47
	532.1, 68	133.9, 53
LSCF1000	528.8, 28	132.3, 42
	532.0, 72	134.0, 58

Figure S1: TGA curves for all samples

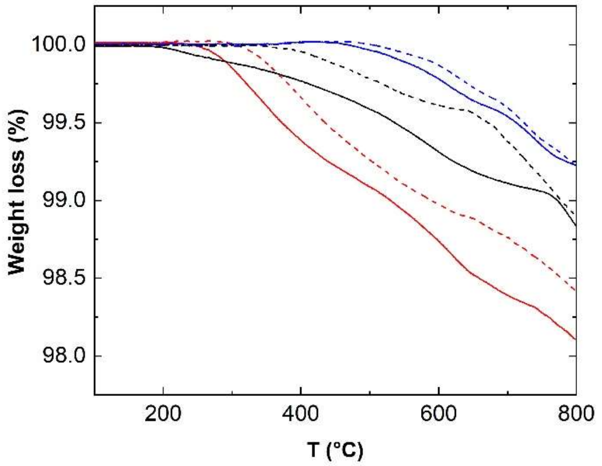

Figure S1. TGA curves calculated from step 4 (see experimental part) for BP-HET (red solid line), LBSCF (red dash line), LSCF900 (black solid line), LSCF1000 (black dash line), BSCF900 (blue solid line) and BSCF1000 (blue dash line).

Image analysis approach: HRTEM results

The powders analysed by means of HRTEM showed an important electron diffraction visible for the presence of several fringes in the direct image space.

The distances between the fringes allow the evaluation of the d_{hkl} values that can be compared with the values obtained by means of XRD to evaluate preferential growth or reciprocal orientation of the crystals.

Even considering regions of the materials showing electron diffraction patterns enough extended to measure a reasonable number of fringes (limiting this way the error of the reading), the variability of the calculated values was huge, making almost impossible a direct identification of the families of planes giving diffraction though a direct comparison with the values obtained by XRD.

It is to note that the issue derived from a typical behaviour of the materials and not from a technical problem of the instrument or of the measurement.

The strategy adopted for obtaining useful information about the materials was, therefore, the following:

1) measurement of several diffraction patterns along a series of at least 10 highly resolved images (around 25 observed d_{hkl} values for sample),
2) grouping the d_{hkl} obtained in small intervals (in our case we considered the intervals 1.65-1.75, 2.02-2.10, 2.22-2.24, 2.27-2.35, 2.38-2.42, 2.68-2.72, 2.78-2.82, 2.89-2.90, larger than 3.00)
3) preparation of the d_{hkl} value distribution curve to define the values observed more frequently in the micrographs.

This way, the considerations on the sample characterization were carried out only considering the most representative values for the system under study.
